# Evaluating the Sensitivity and Specificity of Siemens Clinitest Lateral Flow Test and the Simple AMplification-Based Assay (SAMBA)-2 PCR Test for SARS-CoV-2 Infection

**DOI:** 10.7759/cureus.18319

**Published:** 2021-09-27

**Authors:** Adrian A Boyle, Susie Hardwick, Ben Warne, Chukwuneyem Kosisochukwu Nwuba, Nicholas Brown, Ashley Shaw

**Affiliations:** 1 Emergency Medicine, Addenbrooke's Hospital, Cambridge University Hospitals NHS Foundation Trust, Cambridge, GBR; 2 Infectious Disease, Addenbrooke's Hospital, Cambridge University Hospitals NHS Foundation Trust, Cambridge, GBR; 3 Microbiology, Addenbrooke's Hospital, Cambridge University Hospitals NHS Foundation Trust, Cambridge, GBR; 4 Radiology, Addenbrooke's Hospital, Cambridge University Hospitals NHS Foundation Trust, Cambridge, GBR

**Keywords:** covid-19, emergency department, samba-2, siemens clinitest, pcr test

## Abstract

Introduction

Accurate point-of-care testing for SARS-CoV-2 could quickly identify which patients need to be isolated and improve flow for patients being admitted as an emergency to the hospital. We evaluated two diagnostic tests with shorter turnaround times, the Siemens Clinitest Lateral Flow (Siemens Healthineers AG, Erlangen, Germany) and the Simple AMplification-Based Assay (SAMBA)-2 PCR test against a standard laboratory PCR test.

Methods

We conducted a prospective diagnostic cohort study in a single English emergency department. Adult participants underwent three swabs: the Siemens Clinitest Lateral Flow Test, the SAMBA-2 and a standard laboratory PCR test.

Results

A total of 212 participants were recruited. The sensitivity and specificity of the Siemens Clinitest Lateral Flow Test against the laboratory PCR test was 55.6% (95% CI 30.8-78.5) and 100% (95% CI 98.1-100) respectively. The sensitivity and specificity of the SAMBA-2 PCR test against the laboratory PCR test was 60.0% (95% CI 32.3-83.7) and 100% (95% CI 97.9-100) respectively.

Conclusion

Neither the Siemens Clinitest Lateral Flow Test nor the SAMBA-2 PCR test demonstrated sufficient sensitivity to rule out active SARS-CoV-2 infection. Both tests demonstrated high specificity.

## Introduction

Patients with suspected SARS-CoV-2 infection presenting for emergency care require isolation to prevent nosocomial spread of infection, both to staff and other patients. Current national guidance in the UK is that people should be asked at triage if they have any manifestations of SARS-CoV-2 infection; fever, new continuous cough or anosmia. Any of these features should lead to the patient being cared for in a separate (‘RED’) area of the emergency department. Turn-around times for PCR tests are frequently longer than eight hours, so the type of ward that a patient is subsequently admitted to depends on a clinician estimate of probability of SARS-CoV-2 infection; definite SARS-CoV-2 infection, possible SARS-CoV-2 infection, or unlikely SARS-CoV-2 infection.

Long turnaround times create a number of problems in deciding how to safely isolate people. Most people with any one of these manifestations turn out subsequently not to have SARS-CoV-2 infection, so patients without infection are looked after alongside people with active infection. Furthermore, moving people between wards increases the length of stay and loses continuity of care. Lateral flow tests have been developed to quickly identify patients with, and without, SARS-CoV-2 infection. There is limited and conflicting data about the diagnostic performance of the Siemens Clinitest Lateral Flow test (SCLFT) (Siemens Healthineers AG, Erlangen, Germany). The product information literature states sensitivity and specificity of 98.3% (95% CI 94.1-99.8) and 99.6 (95% CI 98.8-99.9) respectively [1]. An independent evaluation of four lateral flow tests reported the SCLFT sensitivity and specificity to be 54.9 (95% CI 43.4-65.9) and 100 (95% CI 91.5-98.9) [2]. Torres et al. reported a sensitivity of 80% and specificity of over 95% [3]. The WHO has published minimum acceptable sensitivity and specificities of 80% and 97% respectively, below which rapid antigen tests should not be used.

The Simple AMplification-Based Assay (SAMBA-2)-PCR test has a much shorter turnaround time than Public Health England (PHE)-PCR tests. However, there is limited published data about the diagnostic performance of the SAMBA-2 test. The SAMBA-2 test has reported sensitivities and specificities over 90% on reclaimed laboratory samples [4].

A test with a quick turnaround time with high specificity and sensitivity would allow quicker cohorting decisions and reduce bed pressures, by allowing us to isolate confirmed cases and reducing the number of subsequent bed moves. We aimed to evaluate the diagnostic performance of the SCLFT and the SAMBA-2 against standard laboratory PCR results, in patients with possible COVID symptoms. The WHO advises that a sensitivity of greater than 80% is useful for diagnosing SARS-CoV-2 infection, though a lower sensitivity may be acceptable for initial infection control decisions [5].

## Materials and methods

We performed a prospective cohort study in the emergency department of Addenbrookes Hospital, Cambridge, England between January and March 2021. We evaluated this in three stages. In the first stage, we evaluated whether the test could detect obvious cases. Ten tests were taken on people who had a known recent positive COVID PCR test AND were symptomatic (fever, new continuous cough or anosmia). We recruited a convenience sample of patients who had a confirmed diagnosis of COVID on PCR testing within the last three days and were able to consent verbally.

We excluded people from this stage where there was diagnostic doubt about their COVID status or who we were unable to swab because of ongoing clinical care.

Stage 1 confirmed that the SCLFT was sensitive enough to identify obvious cases. We then moved to evaluate this in two separate populations. Stage 2 included people who were asymptomatic and would not have been isolated based on symptoms.

Participants were included in stage 2 if they:

1. Did not have fever, new continuous cough or anosmia

2. Being admitted to hospital

3. Regarded as having a low likelihood of current coronavirus infection by the treating clinician

4. Having a laboratory PCR swab sent but had no available result at the time of enrolment

In the third stage, we recruited patients who had at least one clinical manifestation of fever, new continuous cough or anosmia, but who did not have recent laboratory evidence of confirmed coronavirus infection. This included some people who were being sent home.

Eligible participants were:

1. Clinically thought possible current infection with COVID status recorded in the notes by the treating clinician.

2. Having a laboratory PCR swab sent but had no available result at the time of enrolment.

We excluded prisoners, people who were unable to consent because of confusion, children under the age of 18 and people where there was a known recent swab result within three days.

Each participant was swabbed three times, usually by the same clinician, either nurse or medical student (SH and CN); the SCLFT, SAMBA-2 and the laboratory PCR test. Swabs were taken both from the pharynx and nostril for each test. The laboratory PCR test was treated as the reference standard, these were performed on a Panther Fusion machine. The SCLFT was performed at the bedside following the manufacturer’s instructions, while the SAMBA-2 and PHE PCR tests were sent to different laboratories in the main hospital. A planned subgroup analysis reported performance in symptomatic people, as high viral loads might alter performance characteristics.

Statistical methods

We analysed the data in STATA version 14 (StataCorp LLC, College Station, Texas, USA). We report diagnostic performance with confidence intervals. We were unable to perform an a priori formal sample size calculation based on predictive values as the incidence of COVID-19 varies so quickly. We pragmatically decided that if the SCLFT failed to identify any cases in stage 1, we would not proceed to the second or third stages. For stage 2, on patients with potential COVID symptoms, we planned to recruit around 100 cases, expecting that we would identify around 10 positive PCR cases. For stage 3, we aimed to recruit around 100 cases.

Ethical approval

We were advised that we did not need formal ethical review by our Medical Director (AS) as this was a rapidly required COVID-19 observational study during an NHS Level Four Incident, this was following the instruction of the English Government’s Chief Scientific Officer.

## Results

In stage 1, we recruited nine participants who were thought to have clinically obvious COVID-19, one extra participant was recruited in error. Six out of nine cases were positive compared to the PHE PCR test. This was thought sufficient to proceed to the second and third stages of the study.

In total 212 participants were recruited, three patients declined to participate. Two PHE-PCR and two SAMBA-2 tests were reported as invalid, we missed 10 SAMBA-2 tests. Eighteen tests were positive on PHE-PCR. Sixteen (14.5%) tests were positive in people identified as needing isolation, and two (2%) were positive in people not thought to need isolation at triage, ‘asymptomatic positives’. The two PHE-PCR positive asymptomatic cases were not detected by the SCLFT, and one of these was detected by the SAMBA-2 test. There were no adverse consequences of taking these swabs.

**Figure 1:**
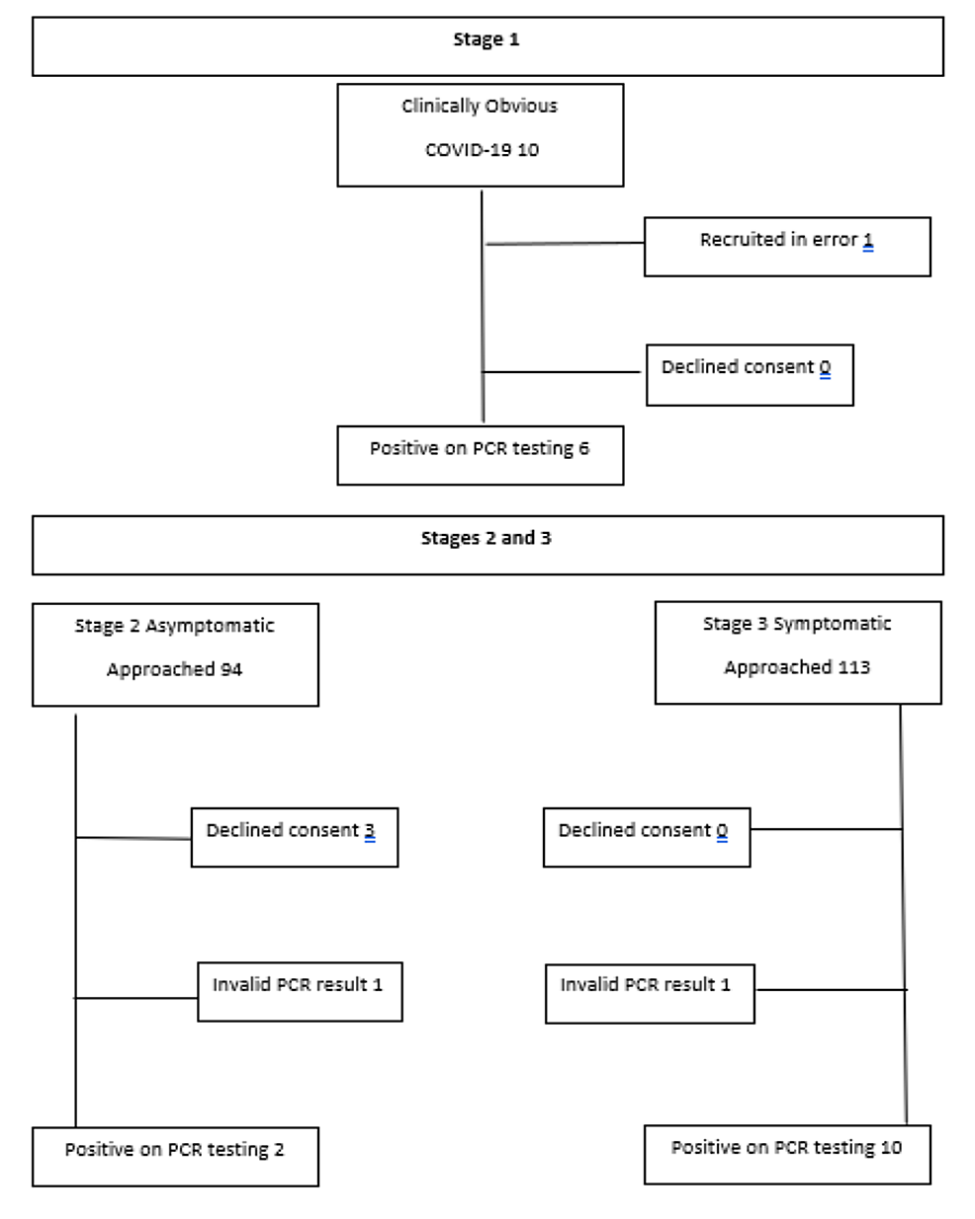
Recruitment of Participants

As a post hoc analysis to further assess the performance of SAMBA-2, we retrospectively compared the results of patients who were tested using both the SAMBA-2 and laboratory PCR platforms, where samples were collected within 24 hours of each other. All specimens were identified using reporting tools in the electronic medical record (EPIC Systems Corporation, Verona, USA). In total, 3098 patients were identified between 1 May 2020 and 15 February 2021. In comparison to the PHE PCR test, the SAMBA-2 was 99.1% (95% CI 98.7-99.4) specific and 70.3% (95% CI 63.6-76.3) sensitive for SARS-CoV-2 infection; sensitivity increased to 73.1% (95% CI 63.5-81.3) when 253 patients with previous positive results in our hospital were excluded from analysis, specificity remained high at 99.5% (95 CI 99.1-99.7). The recruitment of participants is shown in Figure 1.

Table 1 shows the demographic characteristics of the participants. Table 2 shows the turnaround times of the three tests. Table 3 shows the diagnostic performance characteristics of the two tests in all patients. Table 4 shows the diagnostic performance of the two tests in symptomatic patients, both clinically obvious and symptomatic.

**Table 1:** Demographic Characteristics of Participants PHE: Public Health England; PCR: Polymerase chain reaction.

		Number	Number with a positive PCR test	%
Age Group	18-24	10	2	4.7
	25-34	26	2	12.3
	35-44	19	1	8.9
	45-54	34	3	16.0
	55-64	26	4	12.3
	65-74	40	5	18.9
	75 and above	57	2	26.9
	Male Gender	96	8	45.3
	White British Ethnicity	180	17	84.1
	Resuscitation Room Case	13	1	6
	Presenting with at least one symptom of fever, cough or anosmia	111	16	52.4
	COVID recorded in the notes as the dominant diagnosis	14	13	6.6
Final Diagnosis	Trauma and orthopaedics	5	0	1.9
	Medical	156	17	73.6
	Surgical and Gynaecological	25	0	11.7
	Oncological	8	1	4.4
	Other	10	0	4.7
Clinician likelihood of Coronavirus Infection After Evaluation (PHE-PCR positive result)				
	Low	90	1	52.8
	Possible	112	9	42.5
	Definite	10	8	4.7

**Table 2:** Turnaround Times PHE: Public Health England; PCR: Polymerase chain reaction; SAMBA: Simple AMplification-Based Assay.

	Siemens Clinitest Lateral Flow	SAMBA-2	PHE PCR
Number Performed	212	200	212
Number Invalid	0	2	2
Mean Turnaround Time	15 minutes	4:29 hours	16.2 hours
Turnaround Time Range	3-15 minutes	1:48-9:44 hours	4.52-28.32 hours

**Table 3:** Diagnostic Performance of SCLFT and SAMBA-2 against PHE-PCR *The lack of any false positives and low number of positives meant that confidence intervals could not be calculated. PHE: Public Health England; PCR: Polymerase chain reaction; SAMBA: Simple AMplification-Based Assay; SCLFT: Siemens Clinitest Lateral Flow Test.

	Sensitivity	Specificity	Negative Predictive Value	Positive Predictive Value
SCLFT	55.6 (30.8-78.5)	100 (98.1-100)	96.0 (93.5-97.6)	100*
SAMBA-2	60.0 (32.3-83.7)	100 (97.9-100)	96.8 (94.1-98.2)	100*

**Table 4:** Diagnostic Performance of SCLFT and SAMBA-2 against PHE-PCR in symptomatic cases *The lack of any false positives and low number of positives meant that confidence intervals could not be calculated. PHE: Public Health England; PCR: Polymerase chain reaction; SAMBA: Simple AMplification-Based Assay; SCLFT: Siemens Clinitest Lateral Flow Test.

	Sensitivity	Specificity	Negative Predictive Value	Positive Predictive Value
SCLFT	55.6 (21.2-86.3)	100 (96.5-100)	96.2 (92.3-98.1)	100*
SAMBA-2	57.1 (18.4-90.1)	100 (96.3-100)	97.0 (93.3-98.7)	100*

## Discussion

We conducted a prospective clinical cohort study during the second wave of the SARS-CoV-2 pandemic to evaluate both the SCLFT and the SAMBA-2 PCR test. We found that both tests have high specificity and low sensitivity compared to a PHE-PCR test. Neither test achieves the sensitivity threshold of 80% which allows ruling out active infection with Coronavirus. Both tests have excellent specificity. The SCLFT test has a short turnaround time. The relatively long turnaround time for the SAMBA-2 PCR test is caused by multiple factors; batching, the difficulties in getting samples to a remote laboratory, laboratory capacity and IT interfaces. Sensitivity and specificity are the most important performance characteristics if these tests are being used to control infection for hospital admission.

There are a number of important limitations to this study. The study was conducted in a single centre and was a convenience sample, rather than consecutive recruitment. This allowed us to minimise operator error, as most of the swabs were taken by the research team. The sample was slightly enriched with positive cases, this spectrum bias may have increased sensitivity and specificity estimates. The sample size was too small to detect many positive cases, and this led to wide or incalculable confidence intervals for sensitivity and positive predictive values. However, our results had already indicated with the existing sample that both the SAMBA-2 and SCLFT had sensitivities that would make further evaluation futile. Staff recruiting patients were aware of the clinical status of the patient. We did not collect patient data that might influence positivity, such as length of symptoms, but this is indirectly collected by the clinician assessment of potential risk.

Our reference standard can be criticized as solely relying on a laboratory result, but this is the standard we use for allocating patients to an isolation area. It is possible that the cases missed by SAMBA-2 and the SCLFT had low viral loads and may not be actively infectious. Four of the people who had negative SCLFT and positive PCR results had also tested positive more than a week before arrival and these patients may be at a later stage of the disease. Consistent with our clinical experience, COVID-19 was not the dominant diagnosis in most people with a positive PCR test but accompanied several presentations and diagnoses. This data should only be used to make decisions about the deployment of this lateral flow test in this clinical environment. It is uncertain whether these findings can be applied to other areas such as mass population screening.

## Conclusions

These diagnostic performance characteristics are below the 80% threshold advocated by the WHO as effective for diagnosing SARS-CoV-2 infection. There was little difference in performance characteristics between symptomatic and asymptomatic people. These results imply that SCLFT cannot rule out the diagnosis of Coronavirus infection, but can rule a case in. We would not recommend the use of SCLFT for screening asymptomatic patients who are being admitted to hospitals to support infection control procedures in hospitals.
